# Duloxetine and pregabalin in neuropathic pain of lung cancer patients

**DOI:** 10.1002/brb3.1527

**Published:** 2020-01-22

**Authors:** Şule Karabulut Gül, Hüseyin Tepetam, Hakan Levent Gül

**Affiliations:** ^1^ Department of Radiation Oncology Dr.Lutfi Kirdar Kartal Education and Research Hospital University of Health Sciences Istanbul Turkey; ^2^ Sport Sciences Faculty Istanbul Gedik University Istanbul Turkey; ^3^ Department of Neurology Istanbul Maltepe Ersoy Hospital Istanbul Turkey

**Keywords:** cancer, duloxetine, efficacy, neuropathic pain, pregabalin

## Abstract

**Introduction:**

Neuropathic pain occurs in 1% of the population and is difficult to manage. This chronic pain causes psychological distress and impacts patient's quality of life, especially in cancer patients. The aim of this study was to show and compare the efficacy of pregabalin and duloxetine, which are reported in the group of first‐line treatment at European Federation of Neurological Societies (EFNS) guidelines on the pharmacological treatment of neuropathic pain (2010 revision) in lung cancer patients by using visual analogue scale (VAS) and Leeds Assessment of Neuropathic Symptoms and Sign (LANSS).

**Patients and Methods:**

A prospective, randomized, open label, 3 month of study was conducted. A total of 44 patients that were diagnosed with neuropathic pain (14 women and 30 men) were included in the study. Patient's LANSS and VAS values were recorded before treatment. Then, 22 patients undergo pregabalin and 22 patients undergo duloxetine therapy. But due to side effects (dizziness, constipation), two patients had stopped to use pregabalin. Their LANSS and VAS values were recorded after 1 and 3 months of therapy.

**Results:**

When we compare LANSS and VAS scores before treatment, after 1 and 3 months of treatment with pregabalin and duloxetine, a significant decrease was observed in both groups at the 1 and 3 months (*p* < .01). Duloxetine is superior to pregabalin in reducing the LANSS scores when we compare two groups.

**Conclusions:**

Both duloxetine and pregabalin are effective in the treatment of neuropathic pain of lung cancer patients. And as far as we know, this is the first study comparing the efficacy of duloxetine and pregabalin in the neuropathic pain of lung cancer patients.

## Graphical abstract

There are a few number of comparative studies between duloxetine and pregabalin in the treatment of neuropathic pain from Turkey and as well as we know this is the first study comparing these treatments in lung cancer patients. Our aim is to show the efficacy of duloxetine and pregabalin and compare their efficacy in neuropathic pain of lung cancer patients.

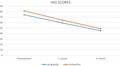

## 1 INTRODUCTION

Neuropathic pain is a common symptom of a group of heterogenic diseases including pain‐related diseases, central or peripheral nervous system injuries, diabetic neuropathy, trigeminal neuralgia, postherpetic neuralgia, and spinal cord injuries. Also, it is common in cancer patients. Neuropathic pain (NeP) has been defined by the International Association for the Study of Pain as pain that is initiated or caused by a primary lesion or dysfunction in the nervous system (Merskey, 1994). NeP often manifests as spontaneous pain (e.g., burning, throbbing, shooting, electric shock sensations), as well as pain that is provoked by stimuli that are normally not painful (allodynia), or that elicit an exaggerated response to a painful stimulus (hyperalgesia; Woolf & Mannion, 1999).

According to the clinical view, neuropathic pain is related to the etiology of various diseases like cancer or diabetes and it is often associated with comorbid conditions (poor sleep, depression, mood disturbances) and a lowered quality of life (QoL; Nicholson & Verma, 2004).

Lung cancer is the most common cancer in the world, and pain is the most common symptom in patients with cancer in general as it also is for lung cancer specifically. Lung cancer patients experience more symptom distress than patients with other types of cancers. This condition is associated with worsening of other symptoms including depression and fatigue, which decreases the quality of life (Nicholson & Verma, 2004).

In conditions of neuropathic pain, neuronal hyperexcitability is the key point but it is not clear that this is peripheral, central, or a combination of both. In diagnosis, the first step is to find out the localization, time, severity and quality of pain, the factors increasing or decreasing the pain and the physical and psychological debility. Neuroanatomic distribution of pain is also important at diagnosis. Classical drugs like antidepressants, anticonvulsants, and antiarrhythmic agents can be used in the treatment of neuropathic pain (Attal et al., 2010).

There are a few number of comparative studies between duloxetine and pregabalin in neuropathic pain from Turkey and as well as we know this is the first study comparing these treatments in lung cancer patients. Our aim is to show the efficacy of duloxetine and pregabalin and compare their efficacy in neuropathic pain of lung cancer patients.

## 2 PATIENTS AND METHODS

A prospective, randomized, open label, 3‐month study was conducted in our Radiation Oncology department to the lung cancer patients receiving radiotherapy and chemotherapy, and a neurologist had included the patients that have neuropathic pain complaints to our study.

Our neurologist had examined the patients for signs and symptoms characteristic of neuropathic pain: “positive” symptoms of neuropathic pain conditions that include both stimulus‐independent (“spontaneous”) and stimulus‐dependent (“evoked”) pain and other symptoms such as tingling (paresthesia) and also for the “negative” signs and symptoms that may be observed which include numbness, weakness, and loss of deep tendon reflexes in the involved nerve territory. So that clinical neurophysiology examines large fibers but is generally not useful in determining the possible involvement of small nerve fibers in neuropathic pain conditions, our neurologist did not perform standard nerve conduction studies to these patients and diagnose was made by the history and neurological examination. A total of 44 patients that were diagnosed as neuropathic pain (14 women and 30 men) were included in the study. Patient's Leeds Assessment of Neuropathic Symptoms and Sign (LANSS) and visual analogue scale (VAS) values were recorded before starting neuropathic pain treatment.

The physicians were asked to fill out the LANSS questionnaire (characterization of the pain true five questions), as well as to perform the two included items for sensory testing: allodynia and altered pin‐prick threshold (Bennett, 2001). Allodynia was judged to be present when pain was elicited by gently stroking a piece of cotton wool over the painful area and when normal sensation was experienced in the control site. Hyperalgesia was judged to be present when pin‐prick testing elicited an exaggerated painful response at the painful site compared with the control site.

Leeds Assessment of Neuropathic Symptoms and Sign scores were recorded before the treatment and at the 1 and 3 months of treatment. For the patients who had a LANSS scale pain score ≥12 means NeP is most probably present. Neuropathic pain was also assessed in a quantitative way by using a visual analogue scale for pain (VAS) offering a score between 0 (no pain) and 100 (very severe pain) to measure pain intensity (Attal et al., 2010; Bennett, 2001; Hawker, Mian, Kendzerska, & French, 2011; Yucel, Senocak, Kocasoy Orhan, Cimen, & Ertas, 2004).

Then, 22 patients undergo pregabalin (group 1) and 22 patients undergo duloxetine (group 2) therapy. pregabalin was used twice a day with a total dose of 300 mg (2 × 150 mg), and duloxetine was used once a day with a dose of 60 mg. Randomization was made due to the admission of the patients to our clinic (1.‐3.‐5…patients to group 1, 2.‐4.‐6…patients to group 2). The study protocol was approved by the ethics committee and made in accordance with the principles of the Helsinki Declaration.

Patients were followed up closely, and their LANSS and VAS values were recorded at the 1 and 3 months of therapy.

Data analysis was made by using SPSS 16.0 for Windows. Descriptive statistics for continuous variables were showed as mean ± standard deviation. Wilcoxon signed rank test was used to evaluate pre‐ and post‐treatment. *p* < .05 were considered as statistically significant.

## 3 RESULTS

A total of 44 patients that were diagnosed with neuropathic pain (14 women and 30 men) were included in the study. Two patients (men) in pregabalin group were excluded from the study due to side effects (dizziness, constipation) at the first week of treatment. Demographic variables are summarized in Table 1.

**Table 1:** Demographic variables

Groups	Number of patients	Gender (F/M)	Age (mean)
Group 1 (pregabalin)	20	7/13	57.45 (33–81)
Group 2 (duloxetine)	22	7/15	58.27 (37–81)

**Figure 1:**
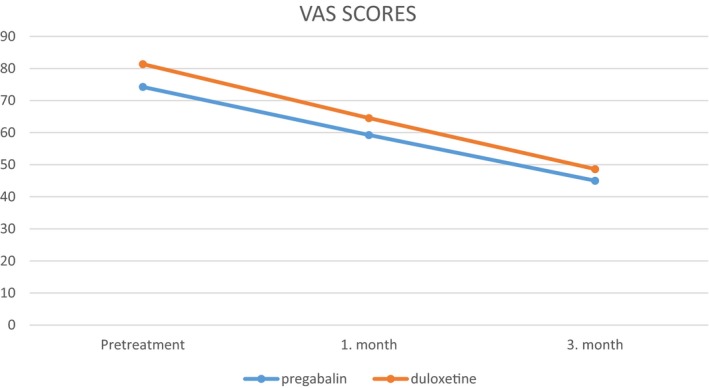
Visual analogue scale (VAS) scores of group 1 and group 2

Group 1 had a mean VAS score of 74.25 before the treatment, 59.25 at the first month of treatment, and 45 at the third month of treatment which are statistically significant (*p* < .001; Figure 1). Group 2 had a mean VAS score of 81.36 before the treatment, 64.54 at the first month of treatment, and 48.63 at the third month of treatment which are statistically significant (*p* < .001; Figure 1).

**Figure 2:**
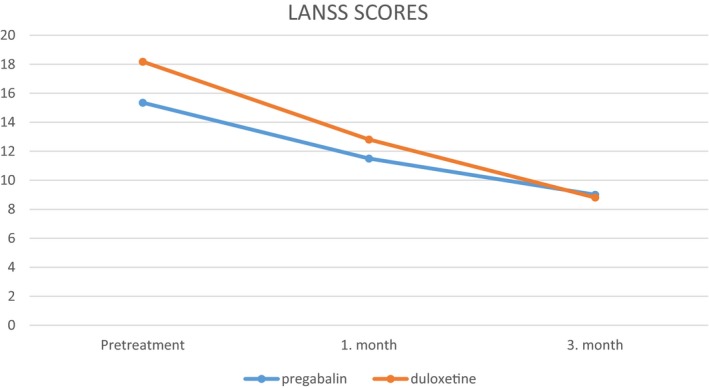
LANNS scores of group 1 and group 2

Group 1 had a mean LANNS score of 15.35 before the treatment, 11.5 at the first month of treatment, and 9 at the third month of treatment which are statistically significant (*p* < .001; Figure 2). Group 2 had a mean LANNS score of 18.18 before the treatment, 12.81 at the first month of treatment, and 8.81 at the third month of treatment which are statistically significant (*p* < .001; Figure 2).

When we compare both groups, pregabalin and duloxetine treatments were found to be effective in decreasing the VAS and LANNS scores, but patients in duloxetine group had a slightly significant decrease in terms of LANNS score at the 3 months of treatment (Figure 2).

## 4 DISCUSSION

In recent years of clinical practice, there is an increasing use of pregabalin and duloxetine in the treatment of neuropathic pain (Namaka et al., 2004). And our study showed that both treatments are quite effective in the neuropathic pain of oncology patients in the short and medium term.

Canadian Pain Society (CPS), European Federation of Neurological Societies (EFNS), Neuropathic Pain Special Interest Group (NeuPSIG), National Institute for Health and Care Excellence (NICE) have all included and recommend pregabalin and duloxetine as the first‐line therapy for neuropathic pain (Cruccu & Truini, 2017).

Pregabalin bind to the calcium channel α2‐δ subunit resulting in decreased central sensitization and nociceptive transmission (Colloca, Ludman, & Bouhassira, 2017). Serotonin norepinephrine reuptake inhibitors (SNRIs) work to block the presynaptic serotonin and norepinephrine transporter proteins, which inhibits the reuptake of these neurotransmitters. Duloxetine inhibits the neurotransmitters equally (Zilliox, 2017).

During the treatment in patients with neuropathic pain; the age of the patient, comorbidities, drug doses, and adverse effects should be evaluated and individualized for each patient. Approximately 60% of cancer patients suffer from neuropathic pain (Pachman, Barton, Swetz, & Loprinzi, 2012).

Mechanism of neuropathic pain in patients with cancer is similar with the other neuropathic pain groups, cancer‐related pain in the etiology of pain (bone invasion, nerve roots and plexus compression, infiltration of the tumor with nerve tissue, vascular infiltration, organ channel blockage, infiltration of fascia, periosteum, and other pain‐sensitive structures, infection and inflammation of the mucous membrane and other pain‐sensitive structures, pain due to the treatment (surgical treatment, chemotherapy, and radiotherapy), and noncancerous pain (trigeminal neuralgia, etc.; Esin & Yalcin, 2014; Fallon, 2013; Pachman et al., 2012; Yoon & Oh, 2018).

When we reviewed the studies that reported the efficacy of pregabalin and duloxetine for the treatment, the dose of 300–600 mg/day for pregabalin and 60 mg/day for duloxetine can often be sufficient and recommended. What we do in our study was to use 300 mg of pregabalin daily and 60 mg of duloxetine daily and to continue the treatment for 3 months during the follow‐up, and we reported similar efficacy as achieved in the literature (Attal et al., 2010; Namaka et al., 2004). Mittal et al. (2011) had made a review of duloxetine and pregabalin in the treatment of painful neuropathy. Tanenberg et al. (2011) had showed the efficacy of duloxetine, pregabalin, and duloxetine plus gabapentin for diabetic peripheral neuropathic pain management in patients with inadequate pain response to gabapentin. As well as we know, these medications are effective in noncancer neuropathic pain.

Mańas et al. (2011) had analyzed the efficacy of pregabalin versus nonpregabalin treatment in patients with cancer‐related neuropathic pain and reported that pregabalin is effective. Mishra, Bhatnagar, Goyal, Rana, and Upadhya (2012) had made a comparative study to show the efficacy of amitriptyline, gabapentin, and pregabalin in neuropathic cancer pain, and showed that pregabalin is more efficient in relieving neuropathic cancer pain and neuropathic symptoms as compared to other antineuropathic drugs. Gul, Erten, Gul, Dama, and Aksoy (2016) had evaluated the efficacy of pregabalin in oncology patients with neuropathic pain and reported that pregabalin was found to be highly effective both in short‐term and mid‐term in oncology patients.

## 5 CONCLUSION

As the result of our study, both pregabalin and duloxetine are effective in reducing the pain severity and intensity of neuropathic pain of oncology patients. It would be more valuable to compare these results with a placebo group but so that the ethic committee did not approve to do so and could decrease the patient's quality of life, we did not use a placebo group.

As new oncologic treatment methods can prolong life, the disease period of oncology patients and the treatment period of the disease with the pain caused by it will be longer. Therefore, it is important to follow‐up the oncology patients with planned treatment protocols, just like the other neuropathic pain groups. But also we recommend a patient‐specific treatment planning for each patient, by considering the comorbidities and side effects of the drugs (e.g., duloxetine or other antidepressant should be used in patients with depression, pregabalin or other antiepileptic drugs should be used in patients with epilepsy) and increase the quality of life.

### 5.1 Limitations

For future trials, we recommend to assess comorbidities (comorbidities like diabetes and radiculopathies, which should also cause neuropathic pain), quality of life, symptoms, and signs with standardized tools and attempt to better define responder profiles to specific drug treatments.

## CONFLICT OF INTEREST

We state that there is no conflict of interest regarding the publication of this paper.

## Data Availability

Also our data that support the findings of this study are openly available in the [Link], [Link], [Link] of this article.
