# Supplementary material for: Duloxetine and pregabalin in neuropathic pain of lung cancer patients
Source: Brain Behav. 2020 Jan 22;10(3):e01527. doi: 10.1002/brb3.1527 (PMC7066365; doi:10.1002/brb3.1527)
Supplement: Supplementary file 1 [file BRB3-10-e01527-s001.docx]

**Table-1. Demographic variables**

| **Groups** | **Number of patients** | **Gender(F/M)** | **Age(mean)** |
| --- | --- | --- | --- |
| Group-1(pregabaline) | 20 | 7/13 | 57.45(33-81) |
| Group-2(duloxetine) | 22 | 7/15 | 58.27(37-81) |
