# Supplementary material for: Duloxetine and pregabalin in neuropathic pain of lung cancer patients
Source: Brain Behav. 2020 Jan 22;10(3):e01527. doi: 10.1002/brb3.1527 (PMC7066365; doi:10.1002/brb3.1527)
Supplement: Supplementary file 2 [file BRB3-10-e01527-s002.docx]

**Figure-1: VAS scores of group-1 and group-2**
